# Genomes of model organisms: know thy tools

**DOI:** 10.1111/j.1462-2920.2008.01656.x

**Published:** 2008-06

**Authors:** Michael Y Galperin

**Affiliations:** National Center for Biotechnology Information, National Library of Medicine, National Institutes of Health Bethesda, MD 20894, USA.

The list of recently completed microbial genome sequencing projects (Table 1) includes genomes of two unicellular eukaryotes, three archaea and a variety of bacteria, including an unusually diverse selection of the *Firmicutes.* The highlights of these sequencing efforts include complete genome sequences of several important model organisms, including the standard laboratory strain *Escherichia coli* DH10B, the model halophile *Halobacterium salinarum* strain R1, the marine cyanobacterium *Synechococcus* sp. PCC 7002 and the unicellular green alga *Chlamydomonas reinhardtii*.

**Table 1:** Recently completed microbial genomes (February–March 2008).

Species name	Taxonomy	GenBank accession	Genome size (bp)	Proteins (total)	Sequencing centre^a^	Reference
New organisms						
*Chlamydomonas reinhardtii*	*Eukaryota, Chlorophyta*	ABCN00000000	∼121 Mbp	14 489	JGI	Merchant *et al.* (2007)
*Monosiga brevicollis*	*Eukaryota, Choanoflagellata*	ABFJ00000000	∼41.6 Mbp	∼9 200	JGI	King *et al.* (2008)
*Candidatus Korarchaeum cryptofilum* OPF8	*Korarchaeota*	CP000968	1 590 757	1 602	JGI	Unpublished
*Thermoproteus neutrophilus*	*Crenarchaeota*	CP001014	1 769 823	1 966	JGI	Unpublished
*Halobacterium salinarum R1*	*Euryachaeota*	AM774415–AM774419	2 668 776 (total)	2 749	MPI Biochem.	Pfeiffer *et al.* (2008)
*Corynebacterium urealyticum*	*Actinobacteria*	AM942444	2 369 219	2 024	Bielefeld U.	Tauch *et al.* (2008)
*Mycobacterium abscessus*	*Actinobacteria*	CU458896CU458745	5 067 172 23 319	4 941	Genoscope	Ripoll *et al*. (2007)
*Cyanothece sp.* ATCC 51142	Cyanobacteria	CP000806–CP000811	5 460 377 (total)	5 304	Wash U.	Unpublished
*Synechococcus sp.* PCC 7002	*Cyanobacteria*	CP000951–CP000957	3 409 935 (total)	3 186	BGI	Unpublished
*Acholeplasma laidlawii*	*Firmicutes*	CP000896	1 496 992	1 380	Moscow Inst. Phys.-Chem.	Unpublished
*Candidatus Desulforudis audaxviator*	*Firmicutes*	CP000860	2 349 476	2 157	JGI	Unpublished
*Finegoldia magna*	*Firmicutes*	AP008971, AP008972	1 797 577 189 163	1 813	RIKEN	Goto *et al*. (2008)
*Heliobacterium modesticaldum*	*Firmicutes*	CP000930	3 075 407	3 000	TGRI	Unpublished
*Leuconostoc citreum* KM20	*Firmicutes*	DQ489736–DQ489740	1 896 614 (total)	1 840	KRIBB	Kim *et al*. (2008)
*Lysinibacillus* (*Bacillus) sphaericus*	*Firmicutes*	CP000817CP000818	4 639 821 177 642	4 771	BGI	Hu *et al*. (2008)
*Thermoanaerobacter pseudethanolicus*	*Firmicutes*	CP000924	2 362 816	2 243	JGI	Unpublished
*Thermoanaerobacter* sp. X514	*Firmicutes*	CP000923	2 457 259	2 349	JGI	Unpublished
*Caulobacter* sp. K31	*α-Proteobacteria*	CP000927, CP000928, CP000929	5 477 872 233 649 177 878	5 438	JGI	Unpublished
*Methylobacterium radiotolerans*	*α*-*Proteobacteria*	CP001001–CP001009	6 899 110 (total)	6 431	JGI	Unpublished
*Methylobacterium* sp. 4–46	*α-Proteobacteria*	CP000943, CP000944, CP000945	7 659 055 57 951 20 019	6 692	JGI	Unpublished
*Cupriavidus taiwanensis*	*β-Proteobacteria*	CU633749CU633750CU633751	3 416 911 2 502 411 557 200		Genoscope	Unpublished
*Leptothrix cholodnii*	*β-Proteobacteria*	CP001013	4 909 403	4 363	JGI	Unpublished
*Polynucleobacter necessarius*	*β-Proteobacteria*	CP001010	1 560 469	1 508	JGI	Unpublished
*Francisella philomiragia*	*γ-Proteobacteria*	CP000937, CP000938	2 045 775 3 936	1 915	JGI	Unpublished
*Shewanella halifaxensis*	*γ-Proteobacteria*	CP000931	5 226 917	4 278	JGI	Unpublished
*Shewanella woodyi*	*γ-Proteobacteria*	CP000961	5 935 403	4 880	JGI	Unpublished
*Leptospira biflexa* strain ‘Patoc 1 (Ames)’	*Spirochaetes*	CP000777, CP000778, CP000779	3 603 977 277 995 74 117	3 600	Institut Pasteur and Monash Univ.	Picardeau *et al*. (2008)
*Leptospira biflexa* strain ‘Patoc 1 (Paris)’	*Spirochaetes*	CP000786, CP000787, CP000788	3 599 677 277 655 74 116	3 787	Institut Pasteur and Monash Univ.	Picardeau *et al*. (2008)
*Thermotoga* sp. RQ2	*Thermotogae*	CP000969	1 877 693	1 819	JGI	Unpublished
New strains						
*Clavibacter michiganensis* ssp. *sepedonicus*	*Actinobacteria*	AM849034	3 258 645	2 943	Sanger institute	Bentley *et al*. (2008)
*Clostridium botulinum* A3 str. Loch Maree	*Firmicutes*	CP000962, CP000963	3 992 906 266 785	3 984	USAMRIID	Smith *et al*. (2007a)
*Clostridium botulinum* B1 str. Okra	*Firmicutes*	CP000939, CP000940	3 958 233 148 780	3 852	USAMRIID	Smith *et al*. (2007a)
*Streptococcus pneumoniae* Hungary19A-6	*Firmicutes*	CP000936	2 245 615	2 155	JCVI	Unpublished
*Ureaplasma parvum* str. ATCC 27815	*Firmicutes*	CP000942	751 679	609	JCVI	Unpublished
*Burkholderia cenocepacia* MC0-3	*β-Proteobacteria*	CP000958, CP000959, CP000960	3 532 883 3 213 911 1 224 595	3 160 2 795 1 053	JGI	Unpublished
*Acinetobacter baumannii* AYE	*γ-Proteobacteria*	CU459137–CU459141	4 048 735 (total)	3 712	Genoscope	Fournier *et al*. (2006)
*Acinetobacter baumannii* SDF	*γ-Proteobacteria*	CU468230–CU468233	3 477 996 (total)	2 975	Genoscope	Fournier *et al*. (2006)
*Escherichia coli* C str. ATCC 8739	*γ-Proteobacteria*	CP000946	4 746 218	4 200	JGI	Unpublished
*Escherichia coli* DH10B	*γ-Proteobacteria*	CP000948	4 686 137	4 126	U. Wisconsin	Durfee *et al*. (2008)
*Escherichia coli* SECEC SMS-3–5	γ-*Proteobacteria*	CP000970–CP000974	5 215 377 (total)	4 913	JCVI	Unpublished
*Haemophilus somnus* 2336	γ-*Proteobacteria*	CP000947	2 263 857	1 980	JGI	Unpublished
*Pseudomonas putida* GB-1	*γ-Proteobacteria*	CP000926	6 078 430	5 409	JGI	Unpublished
*Pseudomonas putida* W619	*γ-Proteobacteria*	CP000949	5 774 330	5 182	JGI	Unpublished
*Xylella fastidiosa* M12	*γ-Proteobacteria*	CP000941	2 475 130	2 104	JGI	Unpublished
*Yersinia pseudotuberculosis* YPIII	*γ-Proteobacteria*	CP000950	4 689 441	4 192	JGI	Unpublished

Sequencing centre names are abbreviated as follows: BGI, Bejing Genomics Institute, Beijing, China; Bielefeld U., Institut für Genomforschung und Systembiologie, Centrum für Biotechnologie, Universität Bielefeld, Bielefeld, Germany; Genoscope, Centre National de Séquençage, Evry cedex, France; Institute Pasteur, Institut Pasteur, Paris, France; JCVI, J. Craig Venter Institute, Rockville, Maryland, USA; JGI, US Department of Energy Joint Genome Institute, Walnut Creek, California, USA; KRIBB, Korea Research Institute of Bioscience and Biotechnology, Daejeon, Korea; Monash Univ., Victorian Bioinformatics Consortium and Department of Microbiology, Monash University, Clayton, Victoria, Australia; Moscow Inst. Phys.-Chem., Research Institute for Physico-Chemical Medicine, Federal Agency of Public Health and Social Development of the Russian Federation, Moscow, Russia; MPI Biochem., Max-Planck-Institute of Biochemistry, Martinsried, Germany; RIKEN, Genome Core Technology Facility, RIKEN Genomic Sciences Center, Yokohama, Kanagawa, Japan; Sanger Institute, The Wellcome Trust Sanger Institute, Wellcome Trust Genome Campus, Hinxton, Cambridgeshire, UK; TGRI, Translational Genomics Research Institute, Scottsdale, Arizona, USA; USAMRIID, United States Army Medical Institute of Infectious Diseases, Fort Detrick, Maryland, USA; U. Wisconsin, Department of Genetics, University of Wisconsin, Madison, Wisconsin, USA; Wash U., Genome Sequencing Center, Washington University School of Medicine, St. Louis, Missouri, USA.

Arguably, the biggest news was sequencing of the genome of *E. coli* DH10B (Durfee *et al*., 2008). Among more than a dozen of *E. coli* strains with completely sequenced genomes, most are pathogenic and only two, MG1655 and W3110, are derivatives of *E. coli* K-12. Strain DH10B was constructed at Douglas Hanahan's lab at Cold Spring Harbor Laboratory (Grant *et al*., 1990) as a derivative of *E. coli* MC1061 designed to serve as a convenient host for cloning and propagation of foreign DNA. Owing to its unusually high transformation efficiency and the ability to maintain large DNA inserts, DH10B became the strain of choice for many genetic engineering tasks and has been extensively used for preparation of mammalian DNA libraries for whole-genome sequencing. Because of this circumstance, the authors were able to replace most of the sequencing with computational analysis of ∼4 million sequence reads collected in the course of the bovine genome sequencing project at Baylor College of Medicine. Bovine BAC DNA preparations were found to contain some (< 1%) DNA contamination from the *E. coli* DH10B host. These DH10B DNA fragments were identified by comparison to the recently updated genomic sequence of *E. coli* K12 strain MG1655 (Riley *et al*., 2006), extracted and assembled into contigs. The genomic finishing phase included identification of the DH10B DNA regions that were absent in the strain MG1655 chromosome and closing the gaps between contigs, which still required some sequencing. After the assembly of *Wolbachia* genomes from *Drosophila* sequence reads by Salzberg and colleagues (2005), this work is another impressive example of extracting useful information on bacterial genomes from the massive amounts of sequence data accumulated by the eukaryotic genome sequencing projects.

The genome sequence of *E. coli* DH10B revealed 226 mutations, a 113 kb tandem duplication and an inversion as compared with the genome of *E. coli* MG1655 (Durfee *et al*., 2008). Surprisingly, the presence of *deoR* mutation in DH10B could not be confirmed, which made the causes of the high transformation efficiency of this strain as obscure as ever before.

In addition to DH10B, two other *E. coli* genomes have been released in March 2008 and will be used for comparative genome analysis. *Escherichia coli* strain SECEC SMS-3–5 was isolated from a toxic metal-contaminated coastal site at Shipyard Creek in Charleston, South Carolina. Surprisingly, this environmental strain is highly resistant to a number of antibiotics, including ciprofloxacin and moxifloxacin, which is obviously a cause for great concern, see http://msc.jcvi.org/e_coli_and_shigella/. *Escherichia coli* C str. ATCC 8739 has an altered outer membrane that lacks the outer membrane porin OmpC and contains only OmpF.

Another important model organism with a recently finished genome is the extremely halophilic archaeon *Halobacterium salinarum* R1. This organism has been first isolated from salted fish in 1920s and has been known under several names, including *Halobacterium halobium*. *Halobacterium salinarum* was used in the famous work of Oesterhelt and Stoeckenius (1971) that discovered bacteriorhodopsin, a 26 kDa protein that comprises the simplest membrane proton pump. Bacteriorhodopsin served as a founding member of a vast family of retinal-binding proteins found in a wide variety of organisms and habitats (Beja *et al*., 2000; Venter *et al*., 2004). Sequencing of the *H. salinarum* R1 genome was performed several years ago, although closing the genome proved impossible at that time owing to the abundance of insertion sequences (Pfeiffer *et al*., 2008). In contrast, *Halobacterium* sp. NRC-1, whose genome has been successfully sequenced (Ng *et al*., 2000), remained taxonomically uncharacterized until 2004 when Gruber and colleagues (2004) showed that it also belongs to *H. salinarum*. Indeed, the recently completed genome sequence of *H. salinarum* R1 proved nearly identical to that of *Halobacterium* sp. NRC-1: most of the observed differences were attributable to the presence of insertion sequences (Pfeiffer *et al*., 2008). Given the significant body of transcriptomic and proteomic data for *H. salinarum* R1 (Aivaliotis *et al*., 2007; Klein *et al*., 2007; Twellmeyer *et al*., 2007), the availability of the genome sequence should make it an even more useful model organism.

The unicellular green alga *Chlamydomonas reinhardtii* is used as a model organism to study photosynthesis, cellular division, intracellular signalling and a variety of other topics. At some point it has even been called ‘the photosynthetic yeast’ (Rochaix, 1995). It has distinct advantages in comparison to higher plants because it is unicellular, haploid and amenable to transformation. It can be grown photoautotrophically or heterotrophically and can be genetically manipulated (Grossman, 2000; 2007). In addition, its genome, as well as the recently released genomes of *Monosiga brevicollis* and *Physcomitrella patens*, is extremely interesting from the evolutionary point of view.

*Monosiga brevicollis* is a representative of a small group of *Choanoflagellates*, unicellular eukaryotes characterized by a single flagellum surrounded by a collar (choane) of microvilli. *Choanoflagellates* are very similar to the choanocytes, specialized cells that are found in several animal phyla, including sponges, the most primitive group of *Metazoa*. This makes them particularly interesting objects for studying the origin of metazoans (King *et al*., 2008). *Monosiga brevicollis genes* contain numerous introns and might be used to clarify the origin of introns and their role in metazoan evolution.

The genome of the model moss *Physcomitrella patens* can be viewed as a bridge from aquatic algae, such as *Chlamydomonas*, to the flowering plants, such as *Arabidopsis*, which appeared at least 400 million years later (Rensing *et al*., 2008).

Another interesting genome that may be important for understanding evolution of life is that of *Candidatus* Korarchaeum cryptofilum, a member of the candidate division *Korarchaeota*. This group does not include any cultivated organisms but, based on the 16S rRNA phylogeny, was proposed to form a separate archaeal phylum, distinct from *Crenarchaeota*, *Euryarchaeota* and *Nanoarchaeota* (hence ‘*cryptofilum*’). Extensive sampling of the Obsidian Pool in Yellowstone National Park in Wyoming allowed collection of sufficiently pure DNA samples to perform the whole-genome sequencing. The completed genome reveals a relatively simple metabolism relying on peptide fermentation. It also confirms that *K. cryptofilum* represents a deep-branching archaeal lineage with limited similarity to *Crenarchaeota*, *Euryarchaeota* or *Nanoarchaeota*, which probably deserves to be considered a separate archaeal phylum.

The three actinobacteria in the current list are all important pathogens: *Clavibacter michiganensis* ssp. *sepedonicus* is a phytopathogen causing the wilt and tuber rot in potato, whereas *Corynebacterium urealyticum* and *Mycobacterium abscessus* are both human pathogens that cause, respectively, urinary tract infections and infections of skin and lungs (Ripoll *et al*., 2007; Tauch *et al*., 2008).

*Clavibacter michiganensis* ssp. *sepedonicus* was first described in 1914 as the causative agent of potato ring rot. It is a close relative of the tomato pathogen *Clavibacter michiganensis* ssp. *michiganensis*, whose genome was sequenced in 2007 (Gartemann *et al*., 2008). However, while *C. michiganensis* ssp. *michiganensis* can survive both as an endophyte and an epiphyte, *C. michiganensis* ssp. *sepedonicus* appears to be limited to the endophytic lifestyle of a potato pathogen (Bentley *et al*., 2008). Genome comparisons suggest a recent evolution of *C. michiganensis* ssp. *sepedonicus*, which resulted in its adaptation to the potato host and included differential gene gain and loss (Bentley *et al*., 2008).

*Mycobacterium abscessus*, first described more than 50 years ago, is a rapidly growing mycobacterium, commonly isolated from soil and water. This organism, formerly known as *Mycobacterium chelonae* ssp. *abscessus* (Kusunoki and Ezaki, 1992), is an important emerging pathogen that causes a variety of human infections, including skin, ear, soft tissue and lung infections (Brown-Elliott and Wallace, 2002; Petrini, 2006). Although it belongs to the group of so-called non-tuberculous mycobacteria, *M. abscessus* can cause a chronic lung infection, similar to tuberculosis, particularly in patients with cystic fibrosis and those undergoing immunosuppressive therapy. *Mycobacterium abscessus* is resistant to many commonly used antibiotics, which makes treatment very difficult.

The marine cyanobacterium *Synechococcus* sp. PCC 7002 was originally isolated in 1961 in Puerto Rico. Owing to its ability to grow fast, either phototrophically or heterotrophically on glycerol, and natural transformability, *Synechococcus* sp. PCC 7002 has become a favourite model organism to study oxygenic photosynthesis (see the Donald Bryant's lab web site http://www.bmb.psu.edu/faculty/bryant/lab/Project/Cyano/ for details).

The second cyanobacterium in the list, *Cyanothece* sp. ATCC 51142, is an aerobic unicellular marine bacterium that is capable of fixing nitrogen and oxygenic photosynthesis (Reddy *et al*., 1993). As nitrogenase, the enzyme responsible for N_2_ fixation, is sensitive to oxygen, photosynthesis and N_2_ fixation cannot occur in the same cell at the same time. *Cyanothece* overcomes this conundrum by using a diurnal cycle: oxygenic photosynthesis and CO_2_ assimilation occur during the day time, while N_2_ fixation occurs during the night (Schneegurt *et al*., 1994). This turnover is apparently regulated by the circadian clock system, which makes *Cyanothece* a good model organism to study the mechanisms of circadian rhythm.

The 1.5 Mbp genome of *Acholeplasma laidlawii* is the largest mollicute genome sequenced to date and the very first one to be sequenced in Russia. Quite appropriately, in Russian street slang, the organism's genus name means something like ‘Why not?’ Like other mycoplasmas, *A. laidlawii* is a common parasite of animals but has been found also in association with plants, in soil, water and raw sewage. It is one of the most frequently identified contaminants of insect and mammalian cell culture. While lacking a cell wall, *A. laidlawii* retains the ability of synthesize fatty acids and glycolipids and does not require exogenous cholesterol, which made it a favourite model organism to study the biophysical properties of biological membranes. *Acholeplasma laidlawii* genome encodes a number of proteins that are not encoded in other mollicutes. These include, among others, components of a signal transduction machinery with two sensory histidine kinases, three response regulators and 14 proteins with diguanylate cyclase (GGDEF) and/or c-di-GMP-specific phosphodiesterase (EAL) domains, which are all missing in previously sequenced mycoplasmal genomes.

*Desulforudis audaxviator* has not yet been cultivated but appears to be a dominant organism in the deep subsurface environment (hence the species name, which means ‘bold traveller’ and comes from Jules Verne's ‘Journey to the Center of the Earth’). This sulfate-reducing bacterium has been has been described so far only in a single poster at the ASM General Meeting in 2006 (Chivian *et al*., 2006) and provisionally assigned to a new genus in the clostridial family *Peptococcaceae*. *Desulforudis audaxviator* was first identified in South African gold mines and detected in almost all fracture fluids emanating from depths ranging from 1.5 to 3.2 km below the surface (Onstott *et al*., 2003). Electron microscopy revealed large cells of up to 4 μm in length. Sequencing the *D. audaxviator* genome was undertaken after analysis of DNA extracted from a borehole water sample collected at 2.8 km depth showed that that more than 93% of that microbial community was *Desulforudis*- type cells. Preliminary genome analysis indicated the ability of *D. audaxviator* to utilize CO and fix N_2_ (Chivian *et al*., 2006). The authors speculate that *D. audaxviator* has retained an ancient mode of metabolism that might sustain life on other planets.

The genome of *Heliobacterium modesticaldum* is the first complete genome sequence from a phototrophic firmicute. This organism is a representative of the family *Heliobacteriaceae*, which unifies spore-forming Gram-positive bacteria that are capable of anoxygenic photosynthesis. The genome of closely related *Heliobacillus mobilis* has been reportedly sequenced by Integrated Genomics, but was never publicly released (Mulkidjanian *et al*., 2006). *Heliobacterium modesticaldum* is a moderately thermophilic anaerobe that was first isolated from a microbial mat in Yellowstone hot spring and grows best at 50–56°C (Kimble *et al*., 1995). This organism is capable of fixing nitrogen and can grow either phototrophically or heterotrophically using pyruvate as a carbon source. The availability of the genome sequence will make *H. modesticaldum* a potential model organism to study the photosynthetic machinery (see the TGRI web site http://genomes.tgen.org/helio.html for more details). It might also help decipher the evolutionary history of anoxygenic photosynthesis, which remains controversial: some authors suggest that heliobacteria possess ancestral photosynthetic machinery (Woese *et al*., 1985; Gupta *et al*., 1999), whereas others believe that heliobacteria acquired it through lateral gene transfer (Mulkidjanian *et al*., 2006). In addition, the ability of *H. modesticaldum* to grow phototrophically at elevated temperatures using N_2_ as nitrogen source makes it attractive for use in biotechnology.

*Finegoldia magna*, formerly known as *Peptostreptococcus magnus*, is a member of the Gram-positive anaerobic cocci, part of the normal human bacterial flora that colonizes skin and mucous membranes of the mouth and gastrointestinal tract (Goto *et al*., 2008). *Finegoldia magna* is an important opportunistic pathogen that is commonly found in clinical samples from infections of soft tissue, bone and joints. The sequenced strain *F. magna* ATCC 29328 was originally isolated from an abdominal wound.

The lactic acid bacterium *Leuconostoc citreum* is used in preparation of various processed foods, such as French cheeses, sauerkraut and pickled cucumbers. Over the past several years, *L. citreum* strains have been isolated from a variety of traditional ethnic foods, including Moroccan soft white cheese; wheat sourdoughs from Southern Italy; pozol, a Mexican traditional fermented corn beverage; traditional fermented milk in South Africa; fermented bamboo tender shoots in North-east India; som-fak, a low-salt fermented fish product from Thailand, and puto, fermented rice cake popular in the Philippines. The sequenced strain *L. citreum* KM20 has been isolated from kimchi, a traditional Korean dish made of fermented napa cabbage, white radish and other vegetables and seasoned with garlic, ginger and hot red pepper (Cho *et al*., 2006). Preliminary analysis of *L. citreum* genome revealed a variety of carbohydrate transporters and glycoside hydrolases, consistent with fermentation of plant material, as well as a mucin-binding protein, consistent with the ability of *L. citreum* to function as a probiotic (Kim *et al*., 2008).

*Lysinibacillus sphaericus* is the recently adopted name of the well-known soil bacterium *Bacillus sphaericus*, some strains of which are pathogenic for mosquito larvae and widely used for insect control (Ahmed *et al*., 2007). As noted earlier, two complete genomes of the insect pathogen *Bacillus thuringiensis*, serovar *konkukian* and strain Al Hakam, were sequenced primarily because of their pathogenicity to humans (Han *et al*., 2006; Challacombe *et al*., 2007). Thus, *L. sphaericus* strain C3-41 is the first complete bacillar genome sequenced solely because of its mosquitocidal properties. The genome paper (Hu *et al*., 2008) offers a detailed analysis of *L. sphaericus* genome and compares it with genomes of six other firmicutes. This comparison reveals a number of significant differences between *L. sphaericus* and both *B. subtilis and B. anthracis*, lending further support to the notion that *L. sphaericus* should be considered a member of a different genus. Remarkably, the closest relative of *L. sphaericus* was *Bacillus* sp. strain NRRL B-14905, isolated from surface waters of the Gulf of Mexico (Siefert *et al*., 2000), whose unfinished whole-genome shotgun sequence (GenBank accession No. AAXV00000000) has been determined at JCVI.

Two more firmicutes with completely sequenced genomes belong to the genus *Thermoanaerobacter. Thermoanaerobacter pseudethanolicus* strain 39E has been isolated from an algal-bacterial mat in Octopus Spring in Yellowstone National Park in Wyoming and initially described as *Clostridium thermohydrosulfuricum* (Zeikus *et al*., 1980). It was later assigned to *Thermoanaerobacter ethanolicus* and recently renamed *T. pseudethanolicus* (Onyenwoke *et al*., 2007). It is a moderately thermophilic (optimal growth at 65°C) anaerobic bacterium that efficiently ferments carbohydrates into ethanol. The ability of *T. pseudethanolicus* to metabolize xylose makes it attractive for use in bioconversion of lignocellulose to industrial alcohol.

*Thermoanaerobacter* sp. X514 is a moderately thermophilic bacterium closely related to *Thermoanaerobacter ethanolicus*. It has been isolated from the deep subsurface environments of Piceance Basin in Colorado (Roh *et al*., 2002). This organism grew optimally at 60°C using molecular hydrogen as an electron donor for Fe(III) reduction. It could also reduce a variety of metals, including Fe(III), Co(III), Cr(VI), Mn(IV) and U(VI) when using acetate, lactate, pyruvate, succinate, glucose and xylose as electron donors. Metal reduction led to the precipitation of various minerals. Thus, reduction of Fe(III) oxyhydroxide (FeOOH) at temperatures ranging from ∼45°C to 70°C led to the production of magnetite Fe_3_O_4_ (Roh *et al*., 2002).

The next two organisms, the α-proteobacterium *Methylobacterium radiotolerans* and the β-proteobacterium *Cupriavidus taiwanensis*, are remarkably similar in their ability to form symbiotic associations with legume roots: they both form root nodules and live there, fixing N_2_ and providing fixed nitrogen to the host plant. At the end of 2007, JGI scientists released the complete genome sequence of the α-proteobacterial methylotroph *Methylobacterium extorquens* strain PA1, a member of the *Rhizobiales* (GenBank accession No. CP000908). That genome has now been followed by genomes of two more members of *Methylobacterium* spp. *Methylobacterium radiotolerans* strain JCM 2831 is a facultative symbiont of legumes that is capable of nodulation and nitrogen fixation, whereas *Methylobacterium* sp. 4–46 apparently is not and will be used for comparative genome analysis.

The nitrogen-fixing β-proteobacterium *Cupriavidus taiwanensis* strain LMG19424 has been isolated from the root nodules of the legumes *Mimosa pudica* and *Mimosa diplotricha* in the southern part of Taiwan and originally named *Ralstonia taiwanensis* (Chen *et al*. 2001). It was subsequently renamed *Wautersia taiwanensis* (Vaneechoutte *et al*. 2004) and, several months later, *Cupriavidus taiwanensis* (Vandamme and Coenye, 2004). It is one of several β-proteobacteria found to be capable of root nodule formation and nitrogen fixation (Moulin *et al*., 2001; Chen *et al*., 2003). The genes responsible for nodule formation and nitrogen fixation were shown to reside on a 0.5 Mbp plasmid. As *C. taiwanensis* is only distantly related to nodule-forming α-proteobacteria, analysis of its genome could define the set of genes that are required for efficient nodulation of plant roots.

The β-proteobacterium *Polynucleobacter necessarius* is an obligate intracellular symbiont of the freshwater ciliate *Euplotes aediculatus*; the organism has not been cultivated outside the host and the host cells cured from *P. necessarius* die after one or two cell divisions (Heckmann and Schmidt, 1987). However, close relatives of *P. necessarius* are found in freshwater habitats all over the world and comprise a large fraction of bacteria in the pelagic zone of surface freshwater (Hahn, 2003). This appears to be a case of a relatively minor sequence divergence between a free-living organism and an obligate endosymbiont (Vannini *et al*., 2007). Complete genome sequence of a free-living *Polynucleobacter* strain QLW-P1DMWA-1 has been released by the JGI a year ago (GenBank accession No. CP000655). The completion of the *P. necessarius* genome offers an opportunity to compare the two and gain important clues on the physiology of this important group of bacteria, as well as the genetic determinants of the intracytoplasmic lifestyle.

The first genome of *Acinetobacter baumannii*, an obligately aerobic bacterium commonly found in soil, water and sewage, as well as in hospital environment, was sequenced in 2007 (Smith *et al*., 2007b). Genomes of two more strains of *A. baumannii* have now been sequenced, an antibiotic-sensitive strain *A. baumannii* SDF, isolated from body lice collected from homeless people living in France (La Scola and Raoult, 2004), and an antibiotic-resistant strain *A. baumannii* AYE.

*Francisella philomiragia*, formerly known as *Yersinia philomiragia*, is a strictly aerobic γ-proteobacterium found in water and fish. It is an emerging pathogen, infecting humans (and fish) with chronic granulomatous disease (Hollis *et al*., 1989; Mikalsen *et al*., 2007). The sequenced strain *Francisella philomiragia* ssp. *philomiragia* ATCC 25017 was isolated from water in the Bear River Refuge in Utah. Genome comparison of *F. philomiragia* and *Francisella tularensis* should help define the pathogenic mechanisms used by these two related bacteria.

The *Shewanella* genome sequencing project at the JGI has released complete genomes of two more marine bacteria, *Shewanella halifaxensis* and *Shewanella woodyi*. *Shewanella halifaxensis* has been isolated from the Emerald Basin, an unexploded ordnance-contaminated marine sediment site near the Halifax Harbor in Nova Scotia, Canada (Zhao *et al*., 2006), together with *Shewanella sediminis* whose complete genome sequence was released by the JGI several months ago (see Galperin, 2007). Like *S. sediminis*, *S. halifaxensis* is capable of metabolizing the explosive agent RDX (hexahydro-1,3,5-trinitro-1,3,5-triazine), which is also known as hexogen, hexolite and cyclonite (see http://pubchem.ncbi.nlm.nih.gov/summary/summary.cgi?cid=8490 for the formula). The periplasmic protein fraction of *S. halifaxensis* transformed RDX almost as well as whole cells, converting it into nitroso derivatives and/or ring cleavage products such as methylenedinitramine (Zhao *et al*., 2008). *Shewanella halifaxensis* is not just an attractive organism for bioremediation of unexploded RDX: it is already hard at work, at least in the Halifax Harbor that gave it its name.

*Shewanella woodyi* is a bioluminescent bacterium that was isolated from seawater and squid ink samples collected from intermediate depth (200–300 m) in the Alboran Sea between Spain and Morocco. These luminous bacteria were unable to ferment sugars but could grow anaerobically using nitrate or nitrite as terminal electron acceptors. The species name was assigned in honour of J. Woodland (‘Woody’) Hastings, a Harvard University professor and a pioneer in studying bacterial luminescence (Makemson *et al*., 1997).
